# Patterns of late spring frost leaf damage and recovery in a European beech (*Fagus sylvatica* L.) stand in south-eastern Germany based on repeated digital photographs

**DOI:** 10.3389/fpls.2015.00110

**Published:** 2015-02-24

**Authors:** Annette Menzel, Raimund Helm, Christian Zang

**Affiliations:** ^1^Ecoclimatology, Department of Ecology and Ecosystem Management, Technische Universitt MnchenFreising, Germany; ^2^Institute for Advanced Study, Technische UniversitätMünchen, Garching, Germany; ^3^Biological and Environmental Sciences, School of Natural Sciences, University of StirlingStirling, UK

**Keywords:** Bayesian analysis, digital photography, multiple change point model, phenology, webcam

## Abstract

Damage by late spring frost is a risk deciduous trees have to cope with in order to optimize the length of their growing season. The timing of spring phenological development plays a crucial role, not only at the species level, but also at the population and individual level, since fresh new leaves are especially vulnerable. For the pronounced late spring frost in May 2011 in Germany, we studied the individual leaf development of 35 deciduous trees (mainly European beech *Fagus sylvatica* L.) at a mountainous forest site in the Bayerischer Wald National Park using repeated digital photographs. Analyses of the time series of greenness by a novel Bayesian multiple change point approach mostly revealed five change points which almost perfectly matched the expected break points in leaf development: (i) start of the first greening between day of the year (DOY) 108–119 (mean 113), (ii) end of greening, and (iii) visible frost damage after the frost on the night of May 3rd/4th (DOY 123/124), (iv) re-sprouting 19–38 days after the frost, and (v) full maturity around DOY 178 (166–184) when all beech crowns had fully recovered. Since frost damage was nearly 100%, individual susceptibility did not depend on the timing of first spring leaf unfolding. However, we could identify significant patterns in fitness linked to an earlier start of leaf unfolding. Those individuals that had an earlier start of greening during the first flushing period had a shorter period of recovery and started the second greening earlier. Thus, phenological timing triggered the speed of recovery from such an extreme event. The maximum greenness achieved, however, did not vary with leaf unfolding dates. Two mountain ashes (*Sorbus aucuparia* L.) were not affected by the low temperatures of -5°C. Time series analysis of webcam pictures can thus improve process-based knowledge and provide valuable insights into the link between phenological variation, late spring frost damage, and recovery within one stand.

## INTRODUCTION

The seasonality of woody plants in cold and temperate climates (or in high and mid-latitudes, respectively) is mainly triggered by the annual course of temperature and photoperiod. Nemani et al. (2003) showed that, in particular, unsuitable (winter) temperatures and sunlight limit vegetation productivity. Consequently, the strategy of deciduous broadleaf tree species is to alternate between a dormant (leafless) period in winter and an active (leaved) growing season in summer. The transition dates, or so-called phenological events, are bud break and leaf unfolding in spring, and leaf coloring and leaf fall in autumn (Schwartz, 2013; Schuster et al., 2013). Spring warming/forcing temperatures are quite well understood as the main triggers inducing bud break and leaf unfolding, making phenology a well suited fingerprint of recent warming (Menzel et al., 2006; Rosenzweig et al., 2008; Menzel, 2013).

After dormancy, trees are more susceptible to challenging environmental impacts such as frost (Rathcke and Lacey, 1985; Sakai and Larcher, 1987; Mayr et al., 2006). Since the likely danger by a too early start of leaf unfolding is being hit by a late spring frost event, the phenotypic plasticity in the timing of this phenological phase provides high adaptive potential (Hosius et al., 2006; Savolainen et al., 2007; Schaberg et al., 2008; Schüler et al., 2012). The specific roles of additional safeguarding strategies to avoid any damage by late spring frost, i.e., fulfillment of a certain amount of winter chilling to break dormancy and/or a certain photoperiod (day length), have been controversially discussed (e.g., Körner and Basler, 2010; Chuine et al., 2010). However, new evidence from multiple twig experiments revealed that chilling is by far more important than photoperiod (Basler and Körner, 2012; Laube et al., 2014; Polgar et al., 2014).

The focus of the current study is late spring frost which restricts tree development (George et al., 1974; Sakai and Larcher, 1987), in particular at altitudinal and latitudinal range limits, and leads to strong economic impacts (Gu et al., 2008). The species studied in this paper, European beech (*Fagus sylvatica* L., hereinafter beech) is the dominant native forest tree species in Central Europe (Leuschner et al., 2006), present under a broad range of climatic conditions from 4 to 13°C mean annual temperature and from 480 to 1360 mm annual sum of precipitation (Kölling, 2007). Its northern and northeastern range limit, e.g., in Poland (Gloning et al., 2013), is likely to be determined by frost in winter and spring (Bolte et al., 2007; see also Kollas et al., 2014). Recent phenological studies have focused on possible impacts of changing climate on its leafing phenology (e.g., Dittmar and Elling, 2006; Donnelly et al., 2006; Delpierre et al., 2009; Caffarra and Donnelly, 2011; Vitasse et al., 2011, 2013; Čufar et al., 2012). Menzel et al. (2006) showed that leafing trends of beech matched the increasing warming pattern in, e.g., mean March temperatures. The cessation of winter dormancy is strongly linked to temperature degree sums of the late winter and spring (von Wuehlisch et al., 1995), and changing photoperiod [Čufar et al., 2012 (controversial); Murray et al., 1989; Heide, 1993; Caffarra and Donnelly, 2011]. Beech as a long-lived late-successional climax species with relatively late leaf unfolding dates, which vary with latitude, longitude, and altitude (Charra-Vaskou et al., 2012), is considered as particularly photosensitive and thus should only respond to warm temperatures once a critical photoperiod has passed (Körner and Basler, 2010). Among comparable deciduous tree species, the temperature response of beech is the smallest (Menzel et al., 2001). Consequently, from a phenological point of view, beech should be less susceptible to late spring frosts. Kramer (1994) and Kreyling et al. (2012b) pointed out that winter buds have a relatively high frost tolerance, which strongly decreases during bud break and leaf unfolding, and the frost resistance increases again after hardening of the leaves, resulting in a short window of up to 2 weeks with high frost sensitivity. Consequently, fresh new leaves of beech will be susceptible to a frost event of -5°C. Equally, Dittmar et al. (2006) reported no significant impacts of frost before leaf unfolding or with temperatures above -3°C on radial growth of beech. Thus, the timing of leaf flushing in relation to minimum temperature is the principal determinant of tolerance to late frost.

Late frost events can occur, e.g., until mid-May in Central Europe, with enormous effects on beech growth, reducing radial growth by more than 90% (Dittmar et al., 2006; Ningre and Colin, 2007). In the worst case, beech individuals lose their leaves entirely and are forced to rebuild them from stored resources. A few papers indicate that the period of refoliation from dormant buds may take up to 36 days (Augspurger, 2009; Awaya et al., 2009); after 2 months the NDVI status prior to frost was achieved again (Kreyling et al., 2012a). Considerable variation was also observed in the percentage of individuals exhibiting frost damage (Augspurger, 2009). Spatial patterns in the damage can be explained by meteorological conditions (altitude, distance to forest edges, canopy cover, or shelter wood), species composition and phenological stages (Petritan et al., 2011; Kreyling et al., 2012b). With climate change, the frequency of cold spells in spring is likely to decrease (Stocker et al., 2013); however, leaf unfolding is also projected to occur much earlier (see Rosenzweig et al., 2008). Consequently, it is not clear whether this has already led, or will lead, to a decreased (Menzel et al., 2003; Scheifinger et al., 2003; Dittmar et al., 2006; Dai et al., 2013) or increased risk of late frost damage (Kramer, 1994; Gu et al., 2008; Inouye, 2008; Augspurger, 2013). Dittmar et al. (2006) reported for southern Germany that the frequency of frost related growth minima increased with altitude. Rigby and Porporato (2008) proposed, using a probabilistic model, that increasing variability in minimum temperatures would increase, whereas increases in mean temperature would decrease frost risk.

There seems to be considerable plasticity among species, populations and individuals (e.g., Augspurger, 2009; Kreyling et al., 2012a,b). Very late leafing types appear to be adapted against a single late frost event (von Wuehlisch et al., 1995; Nielsen and Jorgensen, 2003; Višnjić and Dohrenbusch, 2004; Hufkens et al., 2012) at the expense of a shorter growing season and competitive disadvantages in years without late frost. Populations from warmer origins are generally less tolerant against winter frost (Višnjić and Dohrenbusch, 2004) and late spring frost (Kreyling et al., 2012a) than populations from colder sites. However, the specific phenological behavior matters. In provenance trials, for which a highly significant negative correlation between frost damage and bud break date was revealed (Gömöry and Paule, 2011), the later flushing populations from warmer origins may show the least damage (Chmura and Rozkowski, 2002). Equally, juveniles seem to be more affected by late spring frost due to their (relatively) earlier leaf unfolding (Vitasse et al., 2014). Thus, it is largely phenological differences in bud break which trigger the late frost tolerance of beech populations (Kreyling et al., 2014).

Since tracking of phenology is both labor- and time-intensive, satellite- or webcam-based observation methods have been investigated as possible alternatives (e.g., Nemani et al., 2003; Zhang et al., 2003). Due to the insufficient spatial and temporal resolution of satellite recordings, inexpensive automated digital cameras have become increasingly popular alternatives to the current system of phenological monitoring of ecosystems (Ahrends et al., 2009; Richardson et al., 2009, 2007; Ide and Oguma, 2010; Nagai et al., 2011). Furthermore, analyses of worldwide outdoor webcam images have proved to be useful alternatives to both ground and satellite phenological observation methods (Jacobs et al., 2009; Graham et al., 2010). For late spring frost events, widespread damage could be confirmed for one event in 2010 using satellite remote sensing (Hufkens et al., 2012). Kreyling et al. (2012a) assessed regeneration after the late spring frost in May 2011 in Germany by MODIS NDVI 16 day composites and reported a reset in greening by 7–9 weeks. Using two camera systems, Mizunuma et al. (2013) were able to record the effects of a late frost on canopy greenness, which led to reductions in daily gross primary productivity (GPP). Even differences between individual trees (early flushing ones damaged and recovered, late flushing ones undamaged) could be revealed. Therefore, they suggested the use of spatial information from repeated digital camera images to identify and explain differences between trees in impacts or responses. We studied the May 2011 late frost event in southern Germany and analyzed its pronounced impacts on a beech stand in the Bayerischer Wald National Park as recorded by a phenological camera. In detail, we wanted to fully describe the timing and greening within the annual course of phenology, disturbed by the processes of defoliation and subsequent refoliation based on Bayesian multiple change point modeling. By analyzing the results and distinctive patterns of these models the following questions were answered: (1) How long is the period of recovery till the second green-up? (2) Does the temporal course of the second leafing differ from the first one? (3) Which are the phenological factors influencing damage and recovery? It is certainly a limitation of the study to be restricted to one extreme event captured, however, the intended pattern analysis will foster process based understanding and add to the discussion whether individuals with early or late bud break are more prone to damage.

## MATERIALS AND METHODS

### STUDY SITE, METEOROLOGY, AND LATE SPRING FROST EVENT

The study site Schachtenau (48°56.842′ N, 13°25.237′ E; 807 m above sea level) is located in the Bayerischer Wald (Bavarian Forest) National Park in south-eastern Germany. The climate is humid continental, the mean annual temperature is 5.1°C and the annual sum of precipitation is 1203 mm (data from 2002 to 2012, National Park Administration). From the coarse granite bedrock, acidified sandy-loamy cambisols have developed. Triggered by a wind throw in 1990 and a series of outstandingly warm summers in the 1990s, several mass infestations by the bark beetle *Ips typographus* L. killed a high percentage of Norway spruce (*Picea abies* L.) in the forest stands. Consequently, the vegetation at the study site, which was dominated in 1990 by 100 year old spruce (∼70%) and 70 year old beech with small amounts of other deciduous tree species, shifted in 2011 to standing dead spruce trees, ∼ 25–35 year old spruce and beech regeneration of 6–8 m height as well as formerly suppressed older beech trees of 18–20 m height.

Meteorological data were directly recorded at the Schachtenau tower 50 m above ground, i.e., far above the mean canopy of the stand. Therefore, we additionally used data from an adjacent weather station, located about 1.3 km from the tower inside the forest at two meters height and with an altitudinal difference to the study site of -40 m and similar slope and aspect. We used daily mean, minimum and maximum air temperature values for both stations.

The detrimental late spring frost event occurred on the night of 3rd/4th May 2011 [day of the year (DOY) 123/124] due to a ridge of fast advecting cold polar air masses. The nighttime minimum temperatures dropped to -4.4°C on the tower and to -5.0°C at the adjacent weather station within the forest. Due to a preceding warm and sunny spell with maximum temperatures up to 24°C, beech trees had already started leaf unfolding. The frost severely damaged the fresh leaves and complete hillsides turned brown in this region. Leaves died off, curled up, but mainly stayed on the trees. Trees located at lower elevations than our camera site were less impacted, although temperatures were also below zero.

### CAMERA SET UP AND IMAGE ANALYSIS

**FIGURE 1:**
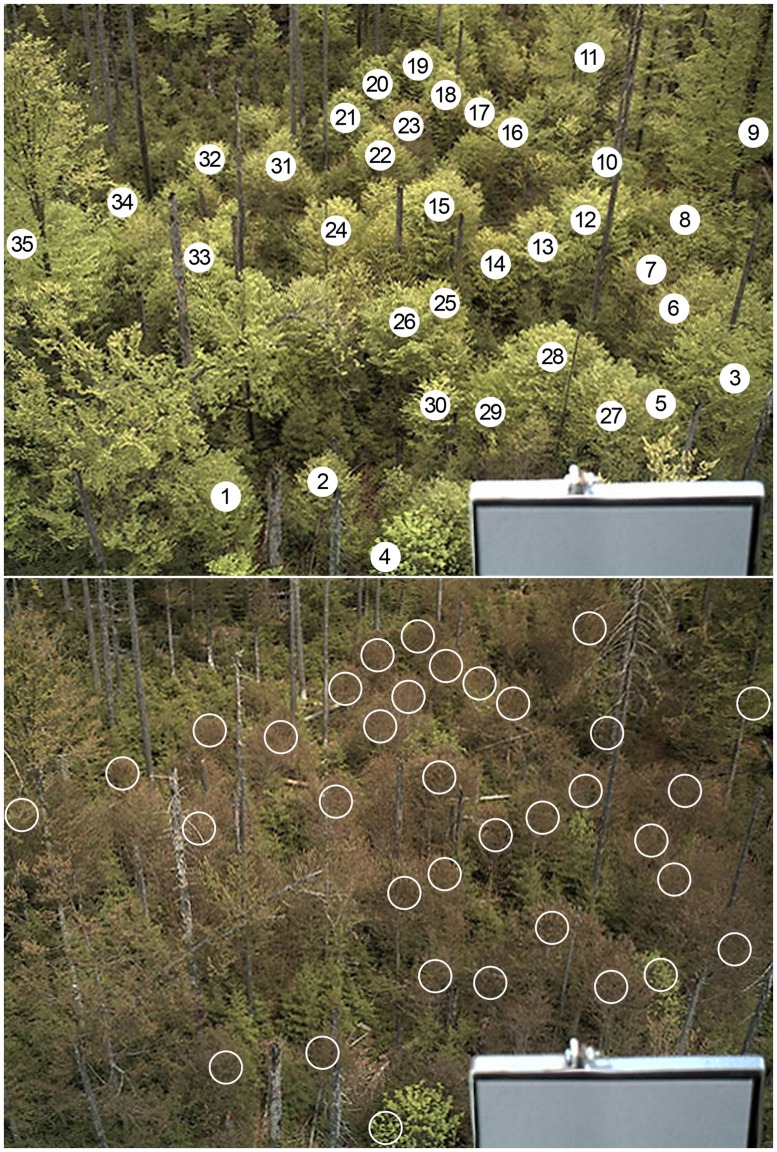
**Sample image of the study site, recorded on May 3rd and 4th, 2011 [day of the year (DOY) 123/124] just before and after the late spring frost event.** White sections indicate regions of interest (ROI). ROI 1–35: canopies of deciduous trees, ROI 36: the whole image excluding the reflectance panel.

Images were taken by a webcam made for security purposes: Mobotix M12 (Mobotix AG, Langmeil, Germany). The camera was installed on the tower at 30 m above ground, with a 350° horizontal and 30° downward vertical angle. In 2011 the camera was automatically controlled by its internal software and took 10 JPEG images per day around midday with an image resolution of 1.2 MP. Exposure and aperture mode as well as the white balance were set to automatic. Due to technical problems the camera was not working on May 25 and July 11 (DOY 145 and 192, respectively). A gray scale reflectance standard [Avian Technologies Fluorilon (TM)] with a reflectance of 18% was installed in the view of the camera; however, calibration of images did not improve the results of this study. The images show deciduous, mainly beech as well as spruce trees; however, this study just focuses on deciduous trees (**Figure 1**).

Image analysis was conducted by defining different regions of interest (ROI) as described by Henneken et al. (2013). Each ROI was manually selected as a circle and represents individual trees (ROI 1–35) and the total stand without the panel (ROI 36; see **Figure 1**). Based on the images during the observation period (DOY 80–210) the positions of the ROI were chosen in such a manner that disturbances from the background of the trees were avoided.

A Python custom script color-split the digital image files sequentially, extracted and averaged the respective color channel information (DN, digital number; red DN, green DN, blue DN). The overall brightness of each ROI (RGB DN) and the proportional values for the green color channel were calculated to minimize the brightness differences between days after the following formula:

$$\begin{array}{c} RGB\,\;\;\,DN=red\,\;DN+green\,\;\;DN\,+\;blue\,\;\;\;DN\\ green\%\;=\;\frac{green\,\;\;DN}{RGB\,\;\;DN} \end{array}$$

The analyses are based on green% which in fact is a proportional value. However, for clarity we hereafter refer to this as greenness or percentage greenness.

### BAYESIAN MULTIPLE CHANGE POINT ANALYSIS

Bayesian multiple change point analysis (Henneken et al., 2013) was used to detect change points in the green% time series of all ROIs. The underlying mathematics and all technical details are given in Dose and Menzel (2004, 2006) and Henneken et al. (2013). The Bayesian multiple change point analysis is a further development of the one-change-point analysis (Dose and Menzel, 2004). For this simplest case the output of traditional methods to describe and segment the time series, e.g., least-square approach, contains a function of two straight lines merging in one change point. The Bayesian alternative also involves change points in the neighborhood of the maximum likelihood change point. Fitting these segments weighted by the respective probabilities of their change points leads to a function which may not consist of straight lines and its associated uncertainty bands. The extension of this method (Henneken et al., 2013) allows detecting multiple change points and calculates probabilities for different numbers of change points. The final model with its associated number of change points is then chosen according to the model probability which is a compromise between the best fit provided, e.g., by an increasing number of change points and the least complexity necessary to describe the data (Ockham’s razor; Garret, 1991).

### IDENTIFICATION OF BEECH PHENOLOGY AND FROST DAMAGE

**FIGURE 2:**
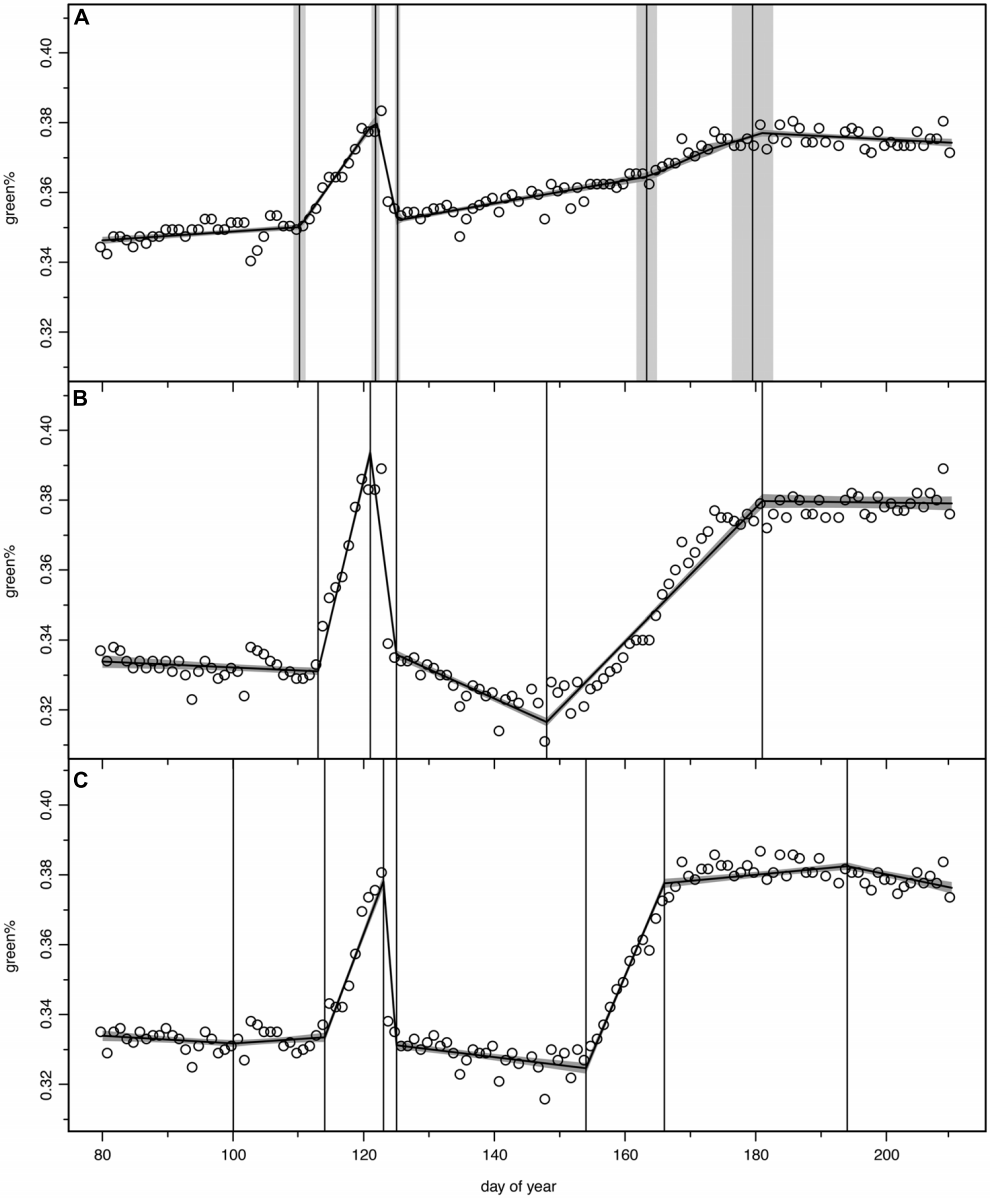
**Bayesian change point models for green% time series, open circles for original camera data, model output as continuous curve with ±1σ uncertainty bands, vertical lines indicate mean and ±1σ uncertainty of the normalized change point probability distributions. (A)** total stand (ROI 36), **(B)** optimal 5-change-point model (ROI 8), **(C)** optimal 7-change-point model, where first and last change point were discarded in the further analysis (ROI 31).

We tested models with 2–7 change points to describe the green% time series of the digital camera between DOY 80 and 210 for each ROI; however, between 5 and 7 change points were identified as the optimal fit. The chosen period allowed a buffer of about 30 days before leafing and extended after the last detected change point, i.e., the fully refoliated canopy at the end of July. **Figure 2A** displays the time series green% of the whole stand (analysis of the full image excluding calibration panel) which was best modeled by the 5 change point option. The vertical bands represent the estimated change points with the mean and standard deviation (σ) of their probability distributions. The uncertainty of the model fit (±1σ) is visualized as a shaded band.

**FIGURE 3:**
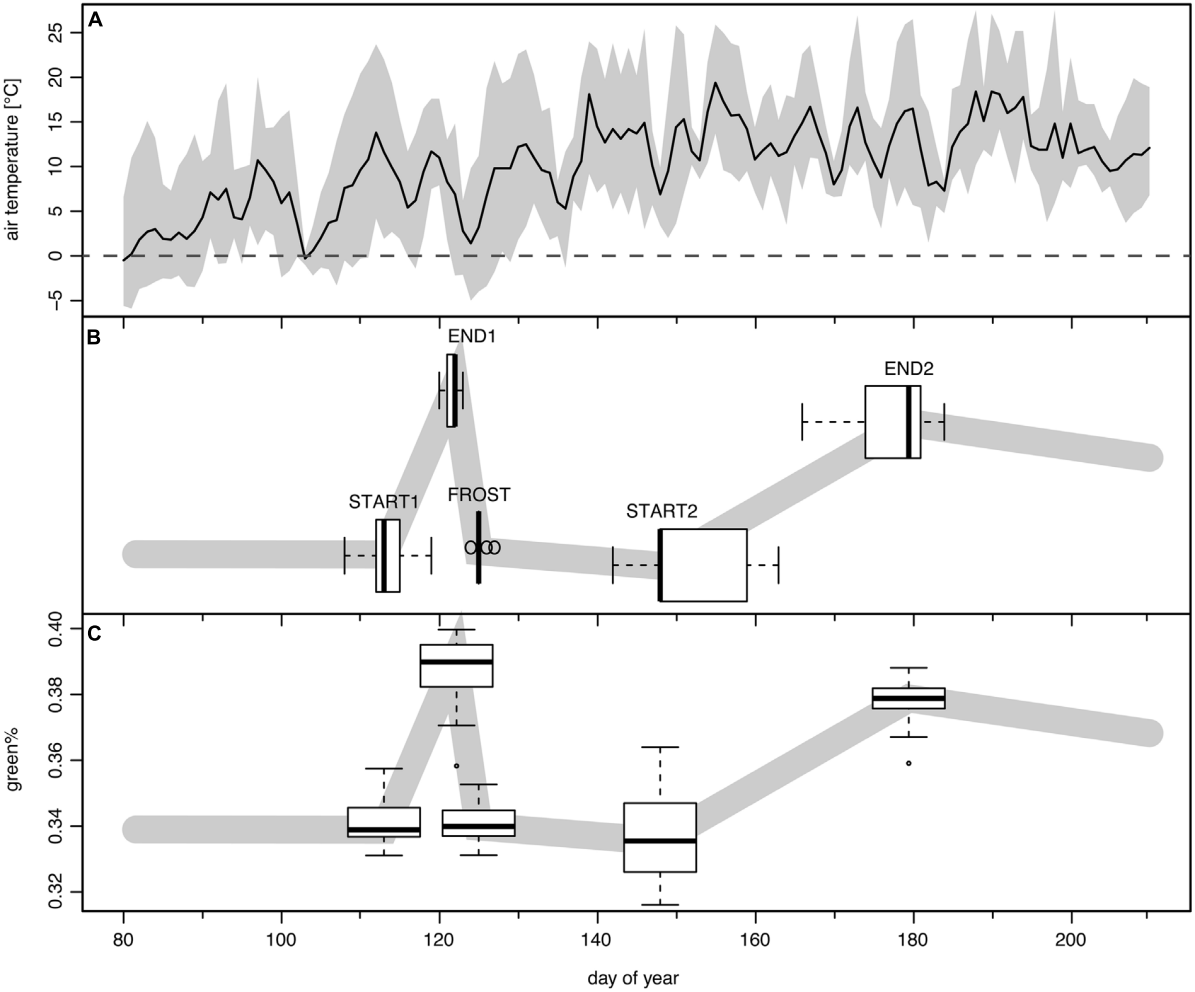
**The frost event of May 3rd to May 4th, 2011 and its modeled impact in time series of green%. (A)** Mean daily (solid line) and range between daily minimum and maximum temperature (shaded band) at the Schachtenau tower. **(B)** Boxplots show the position of the five detected change points as their modeled timing (*n* = 29; see text). **(C)** The boxplots along the schematic green% band indicate the associated modeled green% values (*n* = 29).

The detected change points were assigned to phenological transitions in the course of the spring leaf development, frost damage, and recovery (see **Figure 3**). Change point 1 (hereafter START1) corresponds to the start of leaf unfolding, change point 2 (hereafter END1) is the end of the first greening period (GREENING1) just before the late spring frost event. Change point 3 (hereafter FROST) occurs after the short period of leaf damage (hereafter BROWNING) and mirrors the switch to brown dead leaves. After a longer period of RECOVERY change point 4 (hereafter START2) marks the second leaf unfolding or re-sprouting/St. John’s sprout. Finally change point 5 (hereafter END2) is the transition to completely mature leaves after the second period of greening (GREENING2). The period from START1 to END2 is denoted as TOTAL GREENING.

In the following, START1, END1, FROST, START2, and END2 are given in DOY. GREENING1, BROWNING, RECOVERY, GREENING2, TOTAL GREENING as respective lengths of the periods between the change points are analyzed in days; the respective amount of change (gain/loss) in Δgreen% and the corresponding rates of GREENING1, BROWNING, RECOVERY, GREENING2 as Δgreen% day^-1^. In addition, we calculated the mean green% for the 4 days before START1 (green%_PRESTART_), for DOY 120–123 just before the late frost event (green%_PREFROST_), for DOY 124–143 as the period of RECOVERY (green%_RECOV ERY_), and for the four highest values after DOY 124, corresponding to full leaf status (green%_MAX_).

The relative loss in greenness after the late frost event compared to pre-spring conditions (RESET) is determined as 1 + (green%_PRESTART_ - green%_RECOV ERY_)/(green%_PRESTART_ + green%_RECOV ERY_). RESET ranges between 0 and 2, and is 1 if the amount of green% before START1 and after the frost are identical and <1 if the loss by the late spring frost is less than 100%. Any RESET value of ≥ 1 is connected with full loss.

**Figures 2B,C** display two other examples following the pattern described above, however, with considerable individual variation. The tree in ROI 8 (**Figure 2B**) lost all its fresh leaves since its green% in the recovery period (green%_RECOV ERY_) was even lower than in April (green%_PRESTART_). With the second leaf flush it barely reached the green% before the spring frost event. The canopy in ROI 31 (**Figure 2C**) was best modeled with a 7 change point option and we discarded the first (before leaf unfolding) and the last change point (after leaf maturity). With a high rate of second leaf unfolding (GREENING2) it was able to exceed the green%_PREFROST_ in July.

In total 30 ROIs (29 individual beech trees and the total stand) followed this general pattern of 5 change points. In 10 of these cases where a model with 6 or 7 change points was selected as optimal, we reduced their number to 5, discarding those before START1, after END2 or minor ones during RECOVERY and GREENING2 (see **Figure 2C**, also in contrast to **Figure 6F** for a major change point during RECOVERY). In the other 19 cases the 5 change point model itself was the best or nearly the best choice according to optimization. These 29 single canopies (ROIs) matching the general assumed pattern were further analyzed for relationships between timing of phenological events, timing and loss by the late frost event and timing and amount of recovery of second greening using linear regression. The corresponding p-values were adjusted for multiple testing using the correction by Benjamini and Hochberg (1995). ROIs of the remaining six trees showed very different patterns of leaf unfolding and response to the late spring frost event due to different age, other crown positions as well as other species (2 ROIs). Their responses to the late spring frost event and subsequent recovery are thus described individually, also in order to avoid spurious correlations due to outliers (see **Figure 6**).

## RESULTS

### POSITION OF THE FIVE TRANSITION DATES (CHANGE POINTS) IN RELATION TO THE LATE SPRING FROST

**FIGURE 4:**
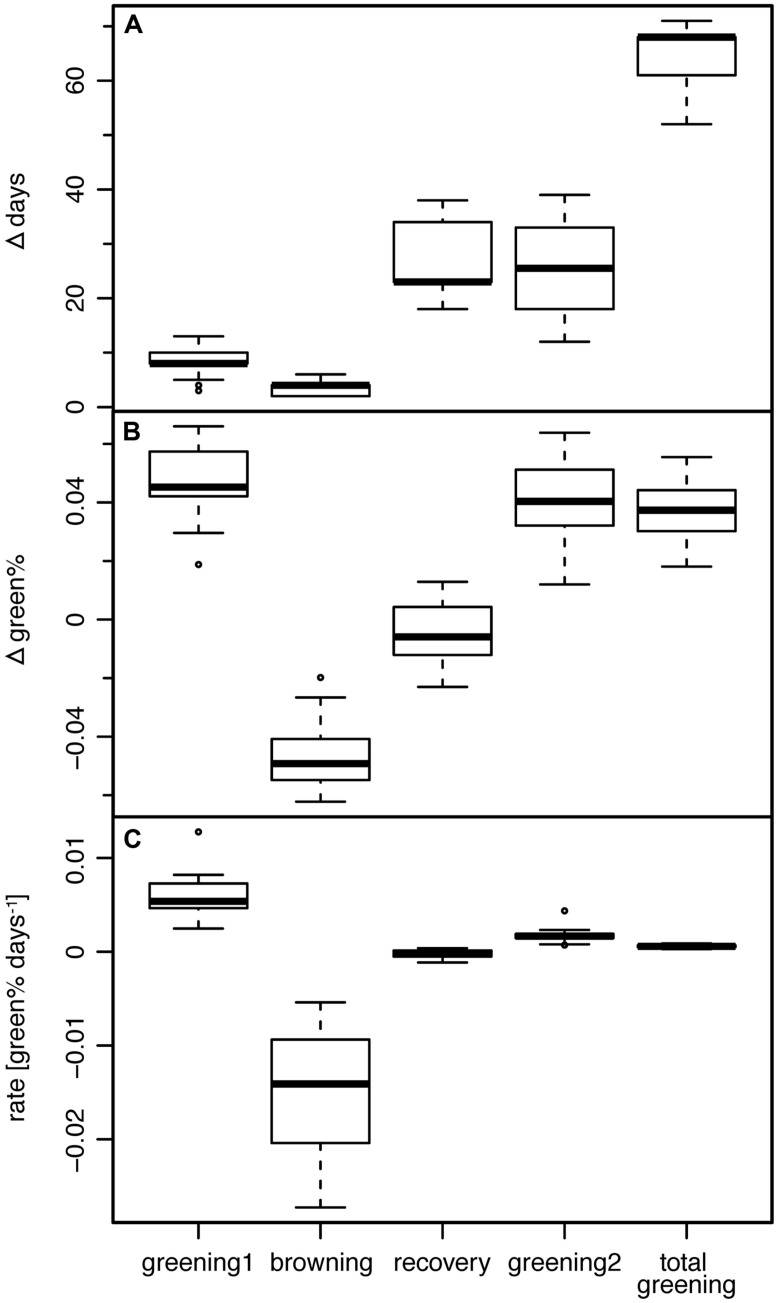
**(A)** Length, **(B)** associated change in green%, and **(C)** corresponding rate of greening of the four periods between change points (see text) and the period TOTAL GREENING (START1 till END2).

Leaf unfolding in 2011 (START1, **Figure 3B**) started on average on DOY 113, with a range of 12 days (DOY 108–119). The corresponding mean green% at START1 was 0.340 and the mean green%_PRESTART_ of ∼0.341 was almost similar (**Figure 3C**). The late spring frost event (overnight between DOY 123/124, **Figure 3A**) was reasonably well captured by the second and the third change point (END1 and FROST) since END1 ranged between DOY 120 and 123 (mean 121.6) and FROST between DOY 124 and 127 (mean 125.1). The best match was DOY 123 (END1) and DOY 125 (FROST) in a few cases where the green% at DOY 123 was maximal and a sharp drop to a local minimum followed. For the other ROIs the Bayesian multiple change point approach revealed a mean time span of 3.5 days between those two events (range 2–6 days, see **Figure 4A**). Thus, the visible frost damage at FROST was always identified as after the frost night (DOY 123/124), whereas the end of the first greening period was assigned to a few days prior to this event.

At END1 after the first period of greening, the trees had achieved a mean green% of 0.388 or mean green%_PREFROST_ of 0.384. This first period of greening lasted, depending on the start of leaf unfolding, on average 8.5 days (between 3 and 13 days) derived from the Bayesian modeling or ∼11 days (between 5 and 16 days) as the interval till the real frost event. Daily leaf assessment in spring 2011 by observers suggested a period of 5–7 days from first leaf till full leaf unfolding; thus, a very high percentage of bud burst should already have occurred by DOY 123.

The damage by the late spring frost which is clearly visible in **Figure 1** was also well captured by the Bayesian modeling. The mean green% at FROST was 0.341 and at START2 was still 0.336, comparable to the mean green%_RECOV ERY_ of 0.338. This means, in terms of visible green in the canopy, that the frost led to a complete reset of leaf development. Green%_RECOV ERY_ (mean 0.338) and green%_PRESTART_ values (mean 0.341) were not significantly different (*p* = 0.14, two-sided Wilcoxon signed rank test). This fact is also captured by the observed mean RESET of 1.004 ranging from 0.985 to 1.024, thus pointing to almost 100% loss. Only for six trees, RESET was ≤0.996.

The following period (RECOVERY) without any visible new greening lasted 18–34 days (mean 26.3 days; **Figure 4A**). The mean rate of greening in this period was as low as -0.00025 Δgreen% day^-1^, i.e., the Bayesian modeling approach really captured the period of brown (**Figure 4B**), damaged or dead leaves without any sign of change in the leaf status (**Figure 4C**).

The second leaf unfolding or re-sprout (START2) was modeled to take place on average on DOY 151.4, ranging between DOY 142 and 161. The following period of second greening lasted 26.3 days on average (12–39 days). On average at DOY 177.5 (DOY 166–184) the crowns reached full maturity again (END2). The timing of modeled START2 and END2 dates had higher standard deviations than START1 and END1, thus the variability between trees was higher than at the beginning of the growing season (see boxplots of **Figure 3B**).

The mean green% of 0.378 which was achieved at the end of the second greening period (END2) was significantly (*p* < 0.0001, two-sided Wilcoxon signed rank test) lower than at END1 (0.388). Only mean green%_MAX_ of 0.384, the four highest values before the end of July, matched the level which was achieved on the 4 days preceding the frost event (mean green%_PREFROST_ 0.384). This means that the loss in green leaves was only just compensated.

The period TOTAL GREENING (START1 till END2) lasted on average 64.3 days (52–71 days) which is a considerable proportion of the total growing season (**Figure 4A**). The mean rate of greening of the first period (0.0061 Δgreen% day^-1^) was significantly higher (*p* < 0.0001, two-sided Wilcoxon signed rank test) than in the second greening period (0.0017 Δgreen% day^-1^).

### PATTERNS IN THE VARIATION OF LEAF DEVELOPMENT

**FIGURE 5:**
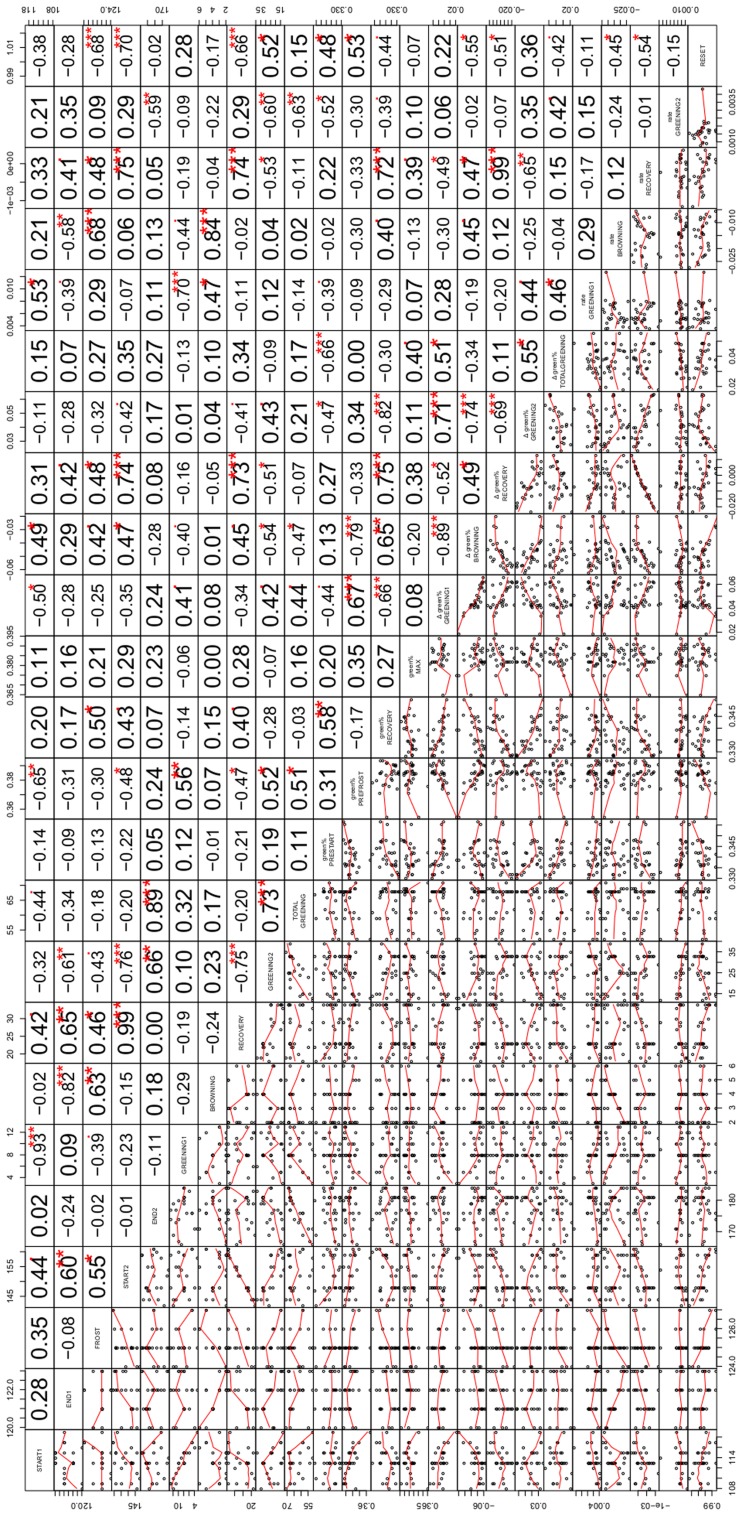
**Correlation matrix of the relationships among the variables examined in the study including miniature scatterplots (lower triangle) and corresponding correlation coefficients (upper triangle).** The variable names on the diagonal refer to both lines and columns and comprise (from left to right) the dates of the five change points (START1, END1, FROST, START2, END2, see Identification of Beech Phenology and Frost Damage), length of corresponding periods (GREENING1, BROWNING, RECOVERY, GREENING2, TOTAL GREENING), related greenness in the characteristic periods of development (green%_PRESTART_, green%_PREFROST_, green%_RECOV ERY_, green%_MAX_), change in greenness during the five periods (Δgreen% GREENING1, Δgreen% BROWNING, Δgreen% RECOVERY, Δgreen% GREENING2, Δgreen% TOTAL GREENING), corresponding rates of greening (rate GREENING1, rate BROWNING, rate RECOVERY, rate GREENING2) and the relative loss after the late spring frost (RESET). Numbers give the respective Pearson correlation coefficients with the significant levels (∙*p* < 0.1, *p < 0.05, **p < 0.01, ***p < 0.001, adjusted for multiple testing).

We systematically tested the correlations between all 24 variables for the 29 modeled ROIs (onset dates at the five change points START1, END1, FROST, START2 and END2, the length of the periods GREENING1, BROWING, RECOVERY, GREENING2, and TOTAL GREENING, the associated changes in green% and the corresponding rates of changes between these change points (except for TOTAL GREENING), and finally the mean green% in the four characteristic periods (green%_PRESTART_, green%_PREFROST_, green%_RECOV ERY_, and green%_MAX)_ as well as the RESET index. Instead of reporting all correlation coefficients and significances (n. s., not significant, *p* < 0.1,^∗^*p* < 0.05, ^∗∗^*p* < 0.01, ^∗∗∗^*p* < 0.001; see **Figure 5** for an overview), we focus on specific questions, e.g., to which variables the onset date (START1) was linked, what might explain variations in RESET, and which factors were associated with a fast and complete recovery after the frost event.

Earlier leaf unfolding of beech (START1) was associated with a higher green% before the frost event (*r* = -0.65^∗∗^, with green%_PREFROST_). Interestingly, individuals with later leaf unfolding showed a higher rate of greening (*r* = 0.53^∗^). Surprisingly, a late start of the first leaf unfolding period was also associated with a later start of the second leaf unfolding period (START2, *r* = 0.44), whereas the two respective rates of greening were not related (*r* = 0.15 n. s.). Later START1 dates were also associated with a shorter total greening period (*r* = -0.44).

The absolute loss of green% by the frost event between END1 and FROST (Δgreen% BROWNING) was strongly linked to green%_PREFROST_ (*r* = -0.79^∗∗∗^) which simply means that if a comparably high leafing was achieved before the frost event a larger amount could be lost. A high significant correlation between Δgreen% GREENING1 and Δgreen% BROWNING (*r* = -0.89^∗∗∗^) points into the same direction. However, the loss of fresh green leaves (RESET) was also negatively associated with START1 (*r* = -0.38 n. s.) which might point to relative differences: the individuals with earlier leaf unfolding experienced a complete loss, whereas late sprouting individuals had lower RESET values pointing to non-maximal losses. In the latter cases of low RESET values the recovery period started (*r* = -0.68^∗∗∗^) and ended (*r* = -0.70^∗∗∗^) significantly later. The more green% the individuals had at the beginning and end of the first greening period, the heavier the relative losses compared to green%_PRESTART_ were (RESET with green%_PRESTART,_ green%_PREFROST_: 0.48^∗^, 0.53^∗^). The higher the RESET, the smaller the rate of recovery (*r* = -0.54^∗^) and the longer the second greening period was (*r* = 0.52^∗^).

All 29 damaged beech trees recovered although the last spring frost event constituted a more or less complete loss of green% (see Position of the Five Transition Dates (Change Points) in Relation to the Late Spring Frost). However, the green%_RECOV ERY_ and green%_PRESTART_ were positively correlated (*r* = 0.58^∗∗^) which means the remaining background greenness in the ROIs before spring leaf unfolding and after the frost event corresponded to a certain degree. A higher green% before the frost event (green%_PREFROST_, *r* = -0.47^∗^) led to a shorter recovery period and consequently earlier second leaf unfolding (*r* = -0.48^∗^). The longer the recovery period was, the higher the gain in green% (*r* = 0.73^∗∗∗^) and rate of greening in this period (*r* = 0.74^∗∗∗^) and the shorter the subsequent second greening period had to be (*r* = -0.75^∗∗∗^).

The overall (net) effect of this extreme event can be assessed by Δgreen% TOTAL GREENING. This was independent from the gain during the recovery period (*r* = 0.11 n. s.), whereas Δgreen% TOTAL GREENING depended on the gain during the first (Δgreen% GREENING 1, *r* = 0.51^∗^) and second greening period (Δgreen% GREENING 2, *r* = 0.55^∗^), which were highly associated (*r* = 0.71^∗∗∗^). Δgreen% TOTAL GREENING was somehow linked to a higher green%_MAX_ at the end of the studied period (*r* = 0.40), since END2 did not match the timing of green%_MAX_.

### INDIVIDUALS NOT MATCHING THE GENERAL PATTERN

**FIGURE 6:**
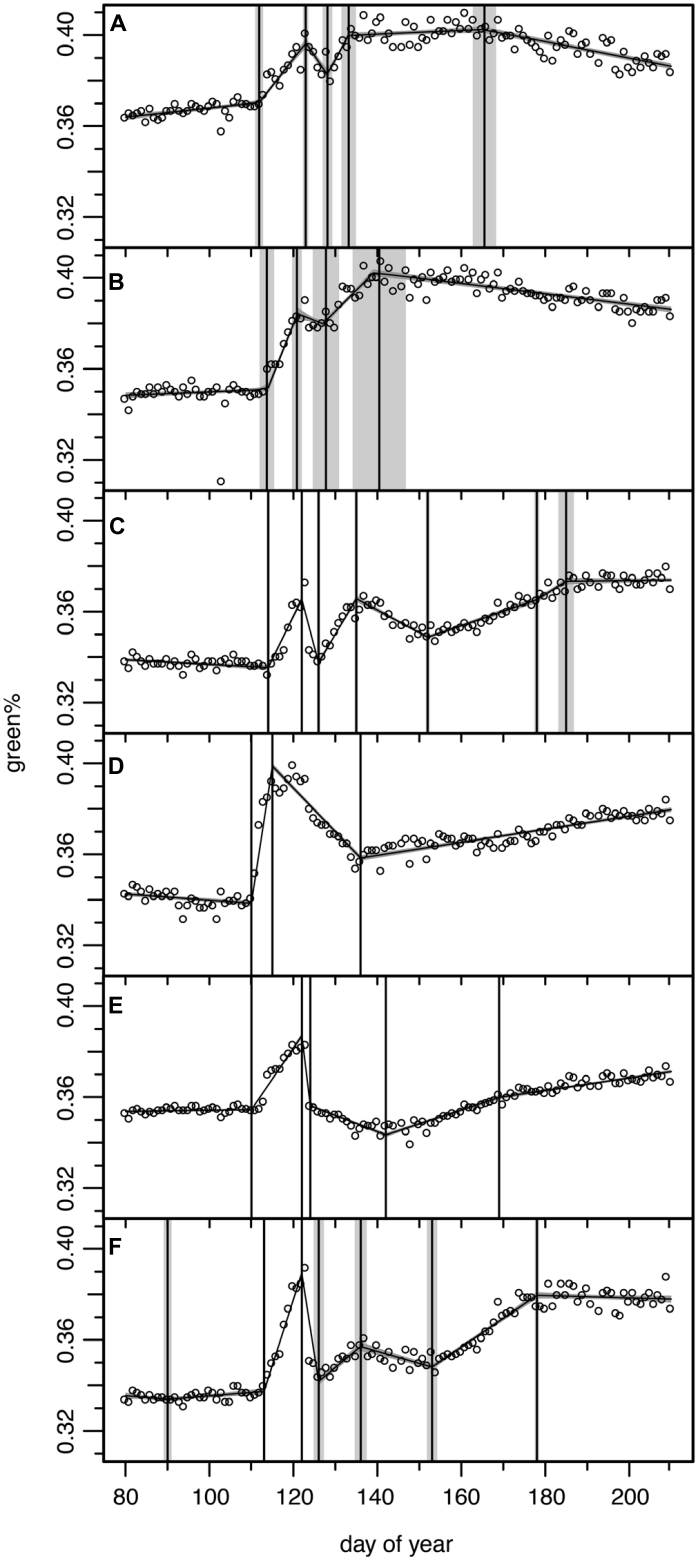
**Results of the Bayesian multiple change point models for six ROIs from six individual trees for which the green% time series did not match the general pattern (see **Figure 2B,C**).** Open circles for original camera data, model output as continuous curve with ±1σ uncertainty bands, vertical lines indicate mean and ±1σ uncertainty of the normalized change point probability distributions. **(A)** ROI 4 with 5 change point model, **(B)** ROI 5 with 4 change point model, **(C)** ROI 7 with 7 change point model, **(D)** ROI 9 with 3 change point model, **(E)** ROI 21 with 5 change point model, **(F)** ROI 29 with 7 change point model (see **Figure 1** for ROIs).

Six individual trees which apparently revealed a much different leaf development, frost damage and re-sprouting pattern were also analyzed by the Bayesian multiple change point approach (see **Figure 6**), but their results were not included in the analysis of the general pattern (see Patterns in the Variation of Leaf Development).

Regions of interest 4 and 5 (**Figures 6A,B**) correspond to two trees which were apparently not or only marginally affected by the late spring frost event. They showed only a slight decrease in green%, reached full maturity early and started to decrease in green% after DOY 166. A careful check at the site revealed that these ROIs corresponded to mountain ashes (*Sorbus aucuparia* L.), one of the few other deciduous tree species present at the study site. This pioneer species is much more frost resistant. In 2011 flowering was observed at DOY 135 and full ripening with its characteristic colorful red berries at DOY 215 (mean phenological onset dates of the German Meteorological Service for comparable altitudes in the Bayerischer Wald National Park). Thus, its early decrease in green% is observed in this period of fruit development.

Regions of interest 9 (**Figure 6D**) corresponds to an older (∼80 year) beech, that formerly has been co-dominant or even suppressed by spruce trees before the bark beetle infestations started in the early 1990s. The ROI focused on its water sprouts in the meso-story, not on its upper crown. In this partly protected area below the main crown the long-wave radiation from the upper parts might have attenuated the low temperatures and the frost damage amounted only to ∼50% of the green% gained before. This ROI then showed a steady but slow recovery never reaching the green%_PREFROST_.

Modeled ROI 21 and 29 time series (**Figures 6E,F**) were difficult to interpret, especially in respect to change points in the recovery period; the beech of ROI 21 also never reached the green%_PREFROST_ level. We could not identify any obvious reason either in the field or in the digital picture explaining these variations in the frost response. ROI 7 (**Figure 6C**) seemed to recover immediately after the frost damage but then lost green% again. Once more, the reason is not clear; perhaps some spruces in the background have distorted the reflectance of this ROI.

## DISCUSSION

The study of an extreme late spring frost event in a (mixed) beech stand in south-eastern Germany, recorded by a phenological camera, allowed a detailed description of the general pattern as well as of the individual variation in spring greening, defoliation, and subsequent refoliation: after a relatively early start of spring leaf unfolding from April 18th till April 29th, an almost complete loss of fresh green leaves after the frost event on the night of 3rd/4th May occurred, a subsequent leafless period of 18–34 days was followed by re-sprouting and leaf maturity. Analyses of these results for 29 individual trees suggested almost no variation of resistance to frost with leaf unfolding dates since the strong advective frost did kill almost all new leaves independent of their age. However, the individual timing of the first leaf unfolding was related to the recovery time after the frost damage and the second leafing period. Thus, in this single case, phenological factors influenced less the damage by, but the recovery from such an event.

Many studies so far have shown that repeated digital pictures of vegetation canopies are suitable for tracking the annual development of vegetation, in particular deciduous trees (Richardson et al., 2007, 2009; Ahrends et al., 2009; Ide and Oguma, 2010; Nagai et al., 2011; Mizunuma et al., 2013; Alberton et al., 2014). In this sense, our study confirms previous experiences. The novelty is the identification of sharp break points in leaf development related to damage and recovery after an extreme late spring frost event based on Bayesian modeling (Henneken et al., 2013).

Extreme events such as droughts are well known to be captured by satellite remote sensing, e.g., NDVI information is used to map their spatial extent and intensity (e.g., Rojas et al., 2011). Only a few studies so far have quantified the impact of a late spring frost event by satellite remote sensing or even repeated digital phenological cameras (e.g., Kreyling et al., 2012a; Mizunuma et al., 2013). Here, our study continuously followed the impacts of this late spring frost damage till the (nearly) full recovery of canopy greenness. We could track individual crown segments of trees, so called ROIs and thus study variation in responses by species and among beech individuals. Even the independent identification of deciduous tree species other than beech based on their frost response captured by the camera would have been possible.

The results of the Bayesian multiple change point model to grasp the timing of all major developmental stages (first greening, frost event, recovery, second greening) were convincing. For the majority of the ROIs, the expected 5 change point model was the best or nearly the best choice according to optimization. Even the solutions with 6 or 7 change points identified the main phenological events correctly. Regular phenological ground observations to validate these change points were only available for the first greening; the data match with the increase in green% of the digital pictures (results not shown). The known timing of the frost (between DOY 123/124) constitutes another possibility to externally validate the results (here FROST damage is modeled between DOY 124 and 127, mean 125.1). However, due to inherent daily variation in the preceding and subsequent green% values, this date could not be nailed down to one single night. The fact that the following recovery period and start of re-sprouting was also linked to a larger individual variability was well captured. The start of re-sprouting was observed from DOY 142 to 161 (mean DOY 151.4 ± 5.8), thus 19–38 days after the frost. The upper end of this span is comparable with the interval provided by Awaya et al. (2009) for late spring frost damage in *Fagus crenata* Blume. If we consider the period between the late frost event and the end of second greening as lost “time” amounting to 43–61 days (mean 54.5 ± 5.0 days), then the results by Kreyling et al. (2012a) for the same frost event of 7–9 weeks based on MODIS NDVI data for southern Germany are matched by our novel method.

The published literature agrees on the fact that the timing of a late spring frost event in relation to the actual phenological development stage would largely determine the damage caused (e.g., Rigby and Porporato, 2008). Briefly, it is the phenological timing that matters and a thorough quantification of the actual risk of late spring frost damage is fundamental since late frost can affect survival, growth, and stem form (e.g., Liu and Muller, 1993; Chaar and Colin, 1999; Dittmar et al., 2006). Studies have reported differences in late frost sensitivity or tolerance between populations in common garden experiments or provenance trials (von Wuehlisch et al., 1995; Višnjić and Dohrenbusch, 2004; Kreyling et al., 2012b). For example, Višnjić and Dohrenbusch (2004) found a strong difference between flushing dates of populations from Italy and Northern Germany. The provenances from Germany flushed about 7 days later and thus were least susceptible to frost (together with provenances from Southeast Europe). In contrast, the early flushing provenances from Italy were most susceptible.

Our study contributes to understanding the individual variations of spring leaf unfolding and subsequent susceptibility to a late frost event. In this regard, only a few publications have reported variations within a stand or population (e.g., Dittmar et al., 2006; Augspurger, 2009; Awaya et al., 2009; Gömöry and Paule, 2011; Kreyling et al., 2012b, 2014). We identified a considerable range of 12 days (DOY 108–119) and a SD of 2.5 days for leaf unfolding among 29 beech trees. Thus, the frost event followed 4–15 days after the start of leaf unfolding. Literature agrees in the general pattern of high frost resistance of buds, a very low resistance around bud break and leaf unfolding, which is then higher again when leaves are fully developed/mature. According to Schwerdtfeger (1981) and Kramer (1994) the individuals with an earlier start of leaf unfolding should have almost been at the stage of increasing frost resistance. Follow-up studies might confirm that timing of a late spring frost event might completely overrule reported relationships of leaf out and damage. If the frost occurs early, only those trees or branches which already have opened their buds will be damaged, whereas with a later, but not as strong frost event older leaves should be less susceptible (see Kramer, 1994).

According to the main association patterns, an earlier start of the growing season might constitute an important (general) fitness parameter. Those individuals had naturally achieved a larger greenness before the frost event; this was due to the larger time span till the frost event. Early leaf unfolding in spring was also significantly related to an earlier start of re-sprouting after the frost and thus a shorter recovery period, pointing to some fitness superiority. We hypothesize that more carbohydrates produced in this 12 days longer period with fresh leaves might enable quicker recovery since they constitute new and potentially easy to relocate resources. However, the green%_MAX_ finally achieved in midsummer was not significantly related to leaf unfolding dates.

However, was there really a significant advantage due to altered frost resistance as suggested by previous findings in the literature? The earlier flushing individuals had not only achieved a larger green% at the time of frost which was then endangered, indeed they also (absolutely) lost more, since we observed almost 100% damage by the late frost event. In contrast to the hypothesis, their damage relative to green%_PRESTART_ was significantly higher than for the late flushing individuals which had very fresh leaves at the time of damage. Does this mean a complete loss fosters a quicker recovery? This surprising result should be interpreted with caution since it is based on a single event and 29 trees only; it might also be the consequence of more brown leaves associated with the early flushing individuals in contrast to late flushing ones with more translucencies for background greenness of the forest floor. In addition, we cannot fully exclude that some trees with low RESET did not yet flush all buds before the frost event and thus continued flushing after the frost, most likely from lateral/basal buds. The latest start date (DOY 119 or 117 for an individual with RESET <1) plus a period of 5–7 days till full leaf unfolding derived from ground observations comes near to the night of the frost (DOY 123/124). The positive relationship of achieved greenness before the frost event and RESET points into that direction. Visual inspection of the Bayesian model description of the green% time series underlined that the accuracy of the modeled START2 dates did not suffer from higher green% during RECOVERY.

Our results could not confirm any variation in frost resistance of fresh beech leaves with age, probably because the frost hit 4–15 days after leaf unfolding and at -5°C it was strong enough to cause a nearly complete loss of new foliage. The ROI 9 (water-sprout of an older beech in the meso-story) did not recover to the same extent like the younger individuals. It is most likely that its shaded position in the meso-story hampered the second greening (see Gressler et al., 2014).

The novel result for the current understanding of advantages of phenotypic differentiation is that phenological timing also triggers the speed of recovery from such an extreme event. More examples which are needed to confirm these first findings will follow in the future with more and more digital phenological cameras set up and regularly operating. In the future not only individual variations in stands, but also differences among populations, e.g., in provenance trials, could be analyzed with Bayesian modeling based on repeated digital camera pictures.

## Conflict of Interest Statement

The authors declare that the research was conducted in the absence of any commercial or financial relationships that could be construed as a potential conflict of interest.
